# Medical Management of Hypophosphatasia: Review of Data on Asfotase Alfa

**DOI:** 10.1007/s11914-025-00906-5

**Published:** 2025-03-18

**Authors:** Kathryn McCrystal Dahir, Nancy S. Dunbar

**Affiliations:** 1 Program for Metabolic Bone Disorders at Vanderbilt, Endocrinology and Diabetes, Division of Endocrinology, Vanderbilt University Medical Center, 8210 Medical Center East, 1215 21St Avenue South, Nashville, TN 37232-8148 USA; 2 Pediatric Metabolic Bone Program, Division of Pediatric Endocrinology, Connecticut Children’s Medical Center, Farmington, CT 06117 USA

**Keywords:** Skeletal dysplasia, Hypophosphatasia, Enzyme replacement therapy, Asfotase alfa

## Abstract

**Purpose:**

Hypophosphatasia (HPP) is a rare, dento-osseous disorder caused by impaired activity of tissue non-specific alkaline phosphatase (TNSALP), a key enzyme in tissue mineralization. This review provides a clinical perspective on the current medical treatment of both children and adults with HPP.

**Recent Findings:**

Dental problems, rickets in children, and osteomalacia in adults are common in HPP. However, disease manifestations in individual patients are exceptionally variable. Recent studies broadened our understanding of HPP symptoms. For example, data showed behavioral health challenges in HPP children, and a large, real-world data set from the Global HPP Registry demonstrated that HPP adults regardless of the time of disease onset exhibit significant disease burden and are broadly affected by non-skeletal impairments, such as pain and chronic fatigue. Treatment for HPP relies on the enzyme replacement asfotase alfa. Small, mostly pediatric trials initially established dosing, safety and efficacy of asfotase alfa, and latest data corroborated the long-term safety and efficacy in both children and pediatric-onset adults. Data from several recent observational studies, including the Global HPP Registry, underscored that asfotase alfa improves physical functions, non-skeletal symptoms such as pain, and quality-of-life (QoL) in adults irrespective of age-of-onset. Clinical use of asfotase alfa is based on prescribing information and evidence-based consensus guidelines. However, recommendations for initiation of therapy are just emerging. Alternatives to asfotase alfa remain limited, but a derivative, efzimfotase alfa, currently undergoes clinical testing.

**Summary:**

Studies in larger HPP patient populations suggest efficacy of enzyme replacement therapy independent of patient age and time of disease onset.

## Hypophosphatasia

### Pathobiology

The dento-osseous bone disorder hypophosphatasia (HPP) results from disease causing variants in the gene *ALPL*, which encodes tissue non-specific alkaline phosphatase (TNSALP) [1]. At least 450 pathogenic *ALPL* variants have been reported [2] (Box 1). Variants can occur *de novo* but far more are inherited in either an autosomal dominant or recessive fashion. Pathogenic variants produce low TNSALP enzyme activity and thus a pathologic accumulation of the TNSALP substrates inorganic pyrophosphate (PPi), pyridoxal 5’-phosphate (PLP), and phosphoethanolamine (PEA). An increase in PPi, an inhibitor of hydroxyapatite formation and mineralization, results in skeletal and dental hypomineralization, a hallmark of HPP. In contrast to PPi, elevated levels of PLP, the physiologically active form of the co-factor vitamin B6, do not directly affect the skeleton but rather negatively impact a range of enzymatic pathways including neurotransmitter synthesis in the brain [3] and carbohydrate metabolism in muscle [4]. Accordingly, perinatal HPP can lead to vitamin B6-depenent seizures and HPP patients of all ages often experience muscle weakness and fatigue. In comparison to PPi and PLP, less is known about the pathophysiology of PEA accumulation in HPP.

The clinical spectrum of HPP ranges from asymptomatic to lethal forms with symptom-onset ranging from prenatal to adulthood [1, 5]. The various clinical forms are typically described by age of symptom onset (perinatal, infantile, childhood, and adult). It is important to note that most forms of HPP have a dental phenotype (commonly the loss of primary dentition before age 4 years), but there is also a separate form of HPP that only affects the teeth and is referred to as odonto-HPP. Variability of clinical manifestations is common in both childhood and adult forms of HPP and even occurs within affected families. Mild disease results more frequently from autosomal dominant inheritance, while severe HPP is more often autosomal recessive, most commonly manifesting from compound heterozygous variants [6].

#### Pediatric

Among pediatric patients, there is a general correlation between onset of symptoms (aside from premature loss of teeth) and severity of clinical disease. Those presenting perinatally or with infantile HPP (before 6 months of age) have high morbidity and mortality due to skeletal and non-skeletal manifestations. Skeletal involvement can be profound with intrauterine fractures, widespread skeletal deformities, and severe rickets. The bones may have a characteristic “soft” or pliable nature due to the inadequate mineralization. Common skeletal features include short, bowed long bones and flat or concave ribs. The reduced mineralization of the rib cage and sternum impairs the mechanical support necessary for normal chest wall function and respiratory mechanics, leading to restricted lung growth and diminished pulmonary compliance. This results in respiratory distress, often requiring prolonged ventilatory support and tracheostomy. Historically, severe respiratory disease was the main contributor to the high mortality rate of untreated perinatal HPP [1, 5]. The more severe forms of childhood HPP tend to present earlier in childhood (but after 6 months) with significant overt skeletal disease and rickets, while less severe forms of childhood HPP generally present later in childhood without rachitic disease [1]. Most childhood HPP cases will also have a history of premature loss of primary teeth. The common pediatric symptoms of HPP can be accompanied by less common or under-recognized manifestations. For example, a recent study in 30 children discovered clinically significant behavioral health challenges in about two-thirds of patients. The most common behavioral findings included sleep disturbance and symptoms of attention deficit hyperactivity disorder, each of which were observed in ≥ 50% of individuals [7].

Two recent investigations within larger patient cohorts have begun to systematically record HPP symptoms (Table 1) which help expand upon what has been gleamed from case reports and case series. A chart review of 50 children with HPP at a single center in Germany reported motor impairment, rickets, and premature loss of teeth as the most common symptoms overall in pediatric HPP [8]*.* A larger study of 151 children using Global HPP Registry data (Box 2) reported bone deformities, muscle weakness, and loss of primary teeth as the most common manifestations [9]. Both studies similarly observed a divergence in symptom severity between the youngest and oldest childhood forms [8, 9]. For example, in the German single center study, perinatal patients (n = 4) were primarily affected by impaired mineralization.

**Table 1:** HPP symptoms observed in large patient cohorts (*n* ≥ 50)

	Pediatric				Adult							
Study Type/Reference	Single center [8]		Global HPP Registry [9]		Global HPP Registry [10]						Global HPP Registry [11]	
Data Source	Chart review		Real world		Real world						Real world	
HPP Onset	Pediatric		Pediatric		Pediatric				Adult		Pediatric/adult	
Number of Patients	50		151		99		114		141		304	
Asfotase alfa treatment	No		No		No		Yes		No		No	
Prevailing Symptoms (%)	Impaired motor skills	78	Bone deformities	45	Dental problems	61	Bone pain	66	Bone pain	53	Pain	67
	Rickets	72	Muscle weakness	34	Loss of primary teeth	58	Dental problems	63	Dental problems	43	Dental problems	54
	Premature loss of teeth	64	Loss of primary teeth	33	Bone pain	46	Body pain	58	Fatigue	23	Skeletal	43
	Musculoskeletal pain	64	Failure to thrive	33	Body pain	46	Poor fracture healing	53	Poor fracture healing	22	Constitutional/metabolic	33
	Craniosynostosis	64	Hypercalcemia, -calciuria, -phosphatemnia	33	Poor fracture healing	33	Fatigue	47	Body pain	22	Muscular	29
	Failure to thrive	62	Rickets	32	Fatigue	31	Loss of primary teeth	47	Muscle pain	19	Renal	15

*HPP* hypophosphatasia

### Box 1: The Global ALPL Gene Variant Database

The Global ALPL Gene Variant Database was established in 2021 by an international, multidisciplinary consortium of HPP experts with the goal to catalog and interpret variants in the ALPL gene, which is crucial in diagnosing and understanding HPP. This consortium includes clinicians, geneticists, and researchers dedicated to the reclassification of ALPL gene variants, particularly those of uncertain significance, to aid in more accurate diagnosis and treatment for HPP patients. The project operates under stringent American College of Medical Genetics and Genomics/Association for Molecular Pathology guidelines for the interpretation of sequence variants and utilizes both literature reviews and functional testing to continuously update and refine variant classifications​. Access to the database is open to the public at https://alplmutationdatabase.jku.at/.

### Box 2: The Global HPP Registry

The Global HPP Registry (https://hppregistry.com/) was initiated in 2014 shortly before regulatory approval of asfotase alfa. It is an international, multicenter, observational study (National Clinical Trial [NCT] number 02306720) designed to collect long-term data on patients with HPP, with the goal of better understanding the natural history, clinical progression, and treatment outcomes of the disease. The registry is managed in collaboration with Alexion, AstraZeneca Rare Disease and multiple academic institutions, ensuring both scientific integrity while fulfilling post-marketing regulatory compliance in several regions. The registry is open to patients of all ages diagnosed with HPP, and it includes data from both untreated and treated patients. Recently published data was based on over 1,200 Global HPP Registry patients and enrollment is ongoing [12]. Enrollment is facilitated by physicians.

(100%), cerebral seizures (100%) and pulmonary abnormalities (100%), while childhood patients mostly suffered from impaired motor skills (69%) and premature loss-of-teeth (69%) [8]. Further, more serious complications such as pulmonary abnormalities occurred in all perinatal patients but only in 65% and 17% of infantile (n = 17) and childhood (n = 29) HPP patients, respectively [8]. Moreover, 9 out of the 16 recorded symptoms had their highest prevalence in perinatal HPP [8]. Data from the Global HPP registry substantiated these findings. Patients presenting with HPP before 6 months (n = 81) generally had a clinical profile of bone deformities (59%), hypercalcemia/hyperphosphatemia (44%), and rickets (43%) [9]. In contrast, older pediatric cases who presented with their first manifestation of HPP after 6 months of age (n = 61) had a clinical profile marked by early loss of primary teeth (62%), gross motor delay (41%), and chronic pain (36%) [9]. The most significant difference in severity was observed for respiratory failure, which had a 23 times higher prevalence in young compared to older patients [9]. Of note, rickets was only present in 16% of the childhood cases presenting after 6 months of age in this study [9]. The information provided by the analysis of these larger cohorts of pediatric HPP patients help clarify the distribution of the skeletal and extra skeletal manifestations and the differing presentation by age.

#### Adult

In contrast to pediatric HPP, manifestations in adults are largely age independent. They often include osteomalacia, fractures and pseudofractures [5]. However, similar to pediatric symptoms it has been difficult to accurately catalogue and quantify adult symptoms based on the historically prevailing case reports or case series. This explains the impact of two recent studies that mined the large dataset collected in the Global HPP Registry for real-world symptoms of adult HPP (Table 1) [10, 11]. The more detailed study compared baseline disease burden in 99 untreated pediatric-onset adults, 114 treated pediatric-onset adults that received asfotase alfa at one point in their life, and 141 adult-onset HPP patients. Untreated and possibly less affected pediatric-onset adult HPP patients chiefly presented with dental problems and early loss of primary teeth but also experienced skeletal and pain manifestations to a lesser proportion than those who were treated [10]. Treated adults with pediatric-onset HPP, which may have experience more pronounced non-skeletal disease, primarily had bone pain, muscle manifestations, dental problems and reduced quality-of-life (QoL) [10]. This suggests that while the symptoms between the two groups are similar, bone pain was more pronounced in the treated and possibly more severe cases of HPP. The significance of bone pain was confirmed by evidence from adult-onset HPP patients, which predominantly manifested in bone pain and dental problems [10]. The other Global HPP Registry study profiled 304 adults with HPP and further supported the prevalence of pain and dental problems in HPP adults [11]. It also showed that, out of 270 analyzed patients, 38% had at least 5 clinical HPP manifestations and 57% had manifestations in at least 3 organ systems, findings that highlight the variability of HPP manifestations [11].

#### Stratification

To stratify the highly variable presentation of HPP, a nosology primarily based on age-of-onset is frequently used [13, 14]. Categorizing HPP by age-of-onset has provided important insights. For example, it showed that the most severe disease occurs in perinatal and infantile patients and is often associated with pulmonary, and neurological complications and high mortality [8, 15]. However, characterizing a highly variable disease in categories is inherently limited. This is exemplified in the significant overlap between the recorded perinatal, infantile, childhood, and adult symptoms [8, 15, 16]. More recently a categorization based on the dominant negative effect of pathological ALPL variants, and their associated disease severity has been proposed and awaits further validation [6]. At least with respect to the medical management of HPP, scoring patients on a spectrum of disease severity, ranging from asymptomatic to severe, might be simpler and more useful than stratification by category. A continuous rather than categorical characterization would not only capture individual patients more accurately but also allow for dynamic monitoring of disease progression as well as disease regression due to therapy.

#### Diagnosis

The clinical presentation of HPP makes its diagnosis challenging and necessitates a thorough and systematic assessment based on clinical, biochemical, radiological and genetic testing. Each patient presents with a unique combination of symptoms from varying organ systems and of varying severity. Many of the common HPP symptoms, such as pain, dental and mobility problems or fractures are not specific for HPP. Therefore, awareness for the disease is important to avoid a delayed diagnosis, and a differential diagnosis that includes HPP is frequently needed. Recently published evidence-based consensus protocols for pediatric and adult patients provide guidelines [5, 17, 18].

## Medical Management of Hypophosphatasia

### Development of the Enzyme Replacement Therapy Asfotase Alfa

The discovery that HPP is caused by decreased TNSALP activity provided a straight-forward rational for enzyme replacement therapy [13, 19]. In a landmark contribution, researchers led by Drs. Crine, Whyte and Milan rationally engineered a recombinant fusion protein composed of a soluble TNSALP, the Fc region of human IgG gamma-1 for Protein A Sepharose purification, and ten acidic aspartate residues for skeletal targeting [20, 21]. Between 2008 and 2010 at least 4 clinical trials were initiated and asfotase alfa tested in patients (Table 2). In 2012, the Federal Drug Administration (FDA) granted a Breakthrough Therapy designation to asfotase alfa. Three years later, in 2015, asfotase alfa received FDA, Canada Health, and European Medicines Agency (EMA) approval for pediatric-onset HPP in the United States, Canada, and Europe, respectively, and Ministry of Health, Labor and Welfare (MHLW) approval for both pediatric and adult-onset HPP in Japan. The drug is manufactured by Alexion, AstraZeneca Rare Disease and has been branded as Strensiq [22].

**Table 2:** Registered* and completed clinical studies in the development of enzyme replacement therapy for HPP

NCT No	Study Type	Start Date	Patients	Aim	Key References
*Asfotase alfa*					
00739505	Interventional, phase 1	2008	Adult, *n* = 6	Safety	
00744042	Interventional, phase 1/2	2008	Pediatric, *n* = 11	Safety, tolerability, pharmacology	[23]
00952484	Interventional, phase 2	2009	Pediatric, *n* = 13	Dose ranging, historic controls	[24]
01163149	Interventional, phase 2	2010	Adult, pediatric, *n* = 19	Dose ranging	[25, 26]
01176266	Interventional, phase 2/3	2010	Pediatric, *n* = 69	Safety, efficacy, pharmacology	[27]
01203826	Interventional, phase 2	2010	Pediatric, *n* = 12	Long-term safety and efficacy	Extension NCT00952484
01205152	Interventional, phase 2	2009	Pediatric, *n* = 10	Safety, efficacy	Extension NCT00744042, [28]
01419028	Observational, retrospective	2012	Adult, pediatric	Natural history of disease (survival)	[29]
02456038	Interventional, phase 2	2014	Adult, pediatric, *n* = 13	Safety, efficacy	TBD
02531867	Interventional, phase 4	2015	Adult, pediatric, *n* = 13	Post-approval	TBD
02797821	Interventional, phase 2	2016	Adult, *n* = 27	Pharmacology	[30, 31]
03418389	Observational	2018	Adult, *n* = 23	Efficacy, PRO	[32–34]
04195763	Observational	2019	Adult, *n* = 50	PROs	TBD
*Efzimfotase alfa*					
04980248	Interventional, phase 1	2021	Adult, *n* = 15	Safety, tolerability, pharmacology	[35]
*Ilofotase alfa*					
05890794	Interventional, phase 1/2	2023	Adult, *n* = 12	Efficacy pilot	TBD

^*^ClinicalTrials.gov

*HPP* hypophosphatasia, *NCT* National Clinical Trial, *PROs* patient reported outcomes, *TBD* to be determined

The pre-approval development of asfotase alfa was accompanied by a single journal publication. In a seminal report, the efficacy of asfotase alfa was demonstrated in 11 infants and young children with life-threatening or debilitating perinatal or infantile HPP [23]. Ten patients completed the study [23]. A 12-months course of asfotase alfa resulted in healing of rickets at 6 months in 9 patients, accompanied by improvement in developmental milestones and pulmonary function as well as markedly improved overall survival [23]. Elevated plasma levels of PPi and PLP diminished with asfotase alfa therapy [23].

### The Treatment Environment

#### Specialists and Patient Referrals

Hypophosphatasia is a rare disease and best managed by experienced specialists that see high volumes of HPP cases and thus have an in-depth knowledge of HPP. The number of specialists in the United States is not known but likely small. A recent Delphi panel to build consensus on assessing HPP severity and disease progression in adult patients included only 31 health care providers and assembled 9 panelists [36]. Most specialists have not received formal training in treating HPP patients but rather developed an interest and expertise during their tenure. The authors believe that the field would greatly benefit from a Rare Bone Diseases Fellowship Program, as it would facilitate seamless transition of knowledge between generations of HPP doctors.

Specialist referrals are common in the early stages of HPP management. Nearly 45% of pediatric cases (n = 18) seen at the Connecticut Children’s Rare Bone Disease program are from family cascade testing. The primary care provider is the most common source of referral (60%) followed by clinical genetics (22%). Similarly, about 30% of adult cases (n = 155) at the Program for Metabolic Bone Disorders at Vanderbilt, stem from cascade family testing, however, referrals come mostly from endocrinology (29%) and clinical genetics (14%), and only occasionally from primary care providers (6%). Referrals from orthopedics (1%) or dentists (1%) are rare, which draws suspicion of undiagnosed patients not receiving care. Notably, about 7% of adult patients have self-diagnosed and seek care directly. The authors’ referral sources might be representative for specialized HPP centers, but there is no systematic documentation or published data on HPP referral sources. Understanding referral sources is warranted because known sources can be educated about HPP. This is important for two reasons. First it facilitates an accurate diagnosis of HPP. Misdiagnoses of HPP are common and can be consequential [5]. For example, in adults a misdiagnosis of osteoporosis can result in initiation of bisphosphonate treatment, which might induce fractures. Second, educated health care providers may recognize the disease at an early stage, helping to avoid unnecessary medical visits and testing, as well as reducing the significant delay between onset of symptoms and diagnosis in patients with HPP [5]. For instance, real-world data from the Global HPP Registry observed a diagnostic delay of 5.7 years in an adult cohort (n = 304) comprising both pediatric and adult-onset patients [11]. A separate analysis from the Global HPP Registry included 269 patients and found less than 1 week diagnostic delay in infants, while a median diagnostic delay of 24.5 years was seen for adults who had a first reported manifestation of HPP before age 18 years and a delay of 3.8 years for those with a first recorded manifestation at or after age 18 years [37].

#### Multidisciplinary Care

Management of patients with HPP includes treatment of acute symptoms, educating about preventive measures, and long-term treatment monitoring. There clearly is a rational for multidisciplinary care. Multidisciplinary care teams were proposed as early as 2017 and thought to be particularly important in managing the transition from pediatric to adult care [15]. Based on an individual patient’s complexity of symptoms and disease trajectory, these teams assemble specialists from a wide range of medical disciplines and coordinate care, an approach thought to benefit outcome. But multidisciplinary care teams are not without challenge. For example, care teams are typically located at centers specialized in HPP care, and for patients the travel distance to those centers might be substantial. This hurdle is amplified by current telehealth regulations that prevent remote patient consultations across state lines. Importantly, sustaining multidisciplinary care teams critically depend on funding and long-term institutional backing.

#### Access to Asfotase Alfa

In the United States, rare disease medicines including biologics such as asfotase alfa are often filled through rare disease pharmacies. These pharmacies provide patient support, adherence outreach, refills, and coordinated delivery of the drug to patients. Latter helps to assure treatment access independent of geographical location.

Medical treatment of HPP is associated with significant costs. Although there are no published health care costs for HPP and pricing for asfotase alfa, which is branded and sold as Strensiq, may vary considerably, retail pricing permits an estimate. The costs for 1 mg Strensiq as subcutaneous solution is $75 (https://www.drugs.com/price-guide/strensiq, accessed October 2024) and therefore annual standard dosing of a 80 kg patients would incur drug costs of approximately $1.8 million. Out of pocket, such costs are unsustainable for a vast majority of patients. Therefore, access to treatment almost always relies on reimbursements and programs that ease patients’ financial burden, including the proposed Medicare Part D limitations on annual out-of-pocket prescription drug costs, are essential for broad access to HPP treatment. However, FDA, Canada Health and EMA regulatory approval of asfotase alfa is restricted to pediatric-onset HPP and most insurance providers follow suit with coverage to these patients. This leaves adult patients often with the burden to prove pediatric-onset or adult off-label treatment.

### Initiation of Asfotase Alfa Therapy

**Fig. 1:**
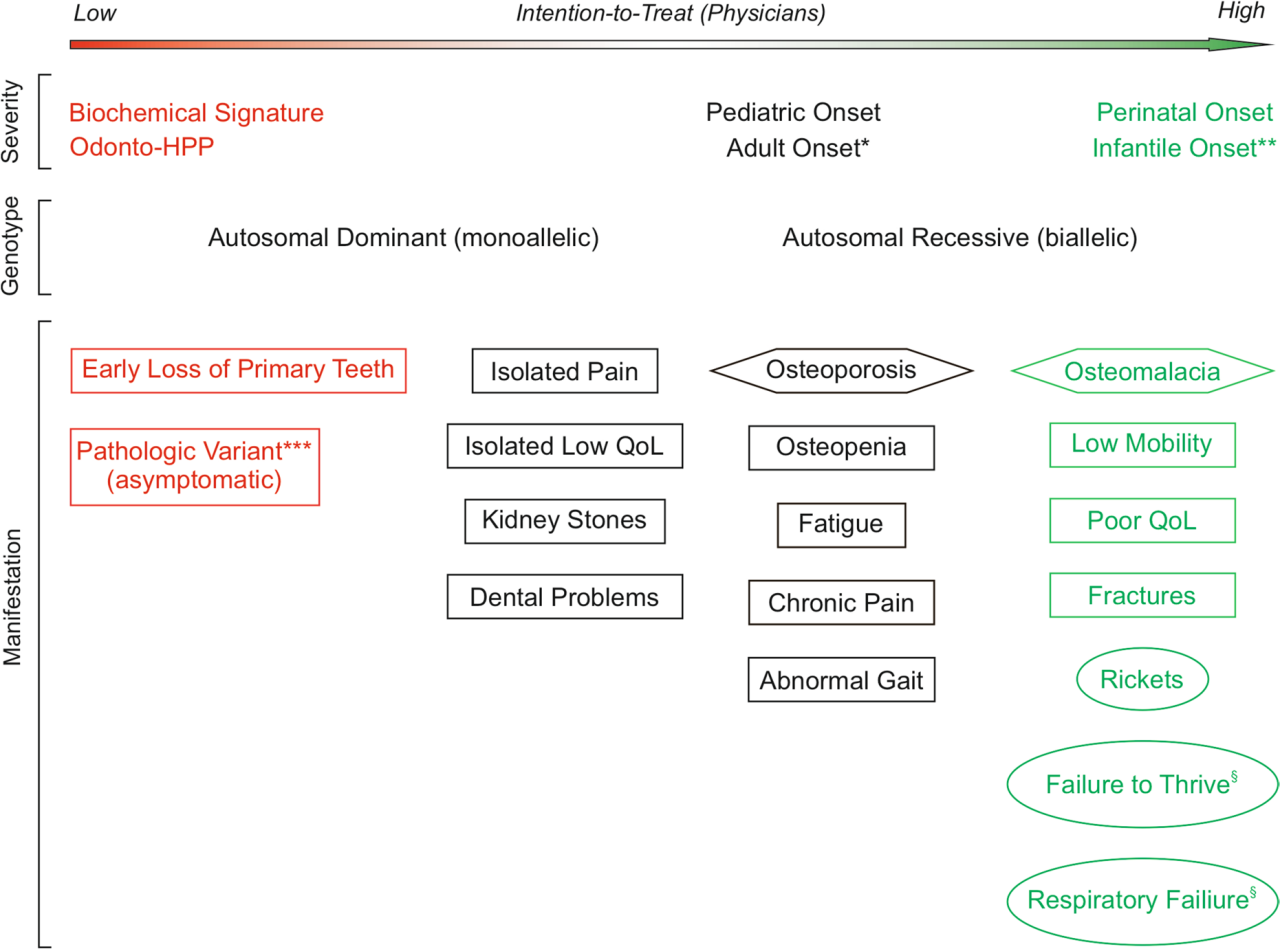
Eminence-based decision making for initiation of asfotase alfa therapy. A total of 26 criteria in three categories are evaluated. Rectangles indicate manifestations seen in both pediatric and adult patients. Hexagons indicate manifestations mostly seen in adults. Ovals indicate pediatric manifestations. Red color: Weak reason for asfotase alfa treatment. Green color: Strong reason for asfotase alfa treatment. *No US regulatory approval of asfotase alfa for adult-onset HPP. **Onset < 6 months of age. ***With or without biochemical signature. ^§^In infancy. HPP, hypophosphatasia

The decision when to initiate treatment remans challenging. At present, there are no evidence-based recommendations available. To provide some guidance and foster a broader discussion [38], the authors identified 26 important criteria in the three categories severity, genotype and manifestations (Fig. 1). For 9 criteria a positive and for 4 criteria a negative treatment recommendation is suggested. However, there is a gray zone for patients comprising manifestations like isolated pain, dental problems, kidney stones or abnormal gait. In these patients the number of manifestations could help to reach a treatment decision. Further, patients play an important role in the decision-making process and intention-to-treat may not always align with medical reasoning, as exemplified by patients requesting treatment based on knowledge of their ALPL variant. Lastly, even if a manifestation, such as chronic pain, would prompt a treatment decision, asfotase alfa may not be a cost-effective treatment.

### Dosing of Asfotase Alfa

The prescribing information for asfotase alfa details the FDA recommended dosage regimen [22] which is weight-based and differentiates between patients with perinatal/infantile and juvenile onset. Perinatal/infantile dosage is 2 mg/kg administered subcutaneously three times per week, or 1 mg/kg administered subcutaneously six times per week. The dose can be increased to 3 mg/kg administered subcutaneously three times per week. Juvenile onset dosage is 2 mg/kg administered subcutaneously three times per week, or 1 mg/kg administered six times per week for both children and adults age 18 and older. No dose escalation is recommended for juvenile onset patients of any age [22].

Over-treatment may excessively lower PPi, potentially increasing the risk of vascular calcifications by diminishing its protective effect against ectopic mineralization. While this risk remains theoretical and lacks strong clinical evidence, factors like pre-existing cardiovascular disease, age, and baseline PPi levels could play a role. One reassuring case report demonstrated that 8 months of asfotase alfa showed no evidence of vascular calcifications or other concerning ectopic mineralization [39]. A challenge in safety monitoring is that PPi assays are not commercially available, and in vitro, asfotase alfa in a patient’s serum can continue to dephosphorylate PLP into pyridoxal, complicating assay results. Regular monitoring of biochemical markers such as calcium and phosphate is recommended during treatment.

### Efficacy of Asfotase Alfa Therapy

The efficacy of asfotase alfa was first demonstrated in clinical trials in children [23, 27]. Pharmacokinetic and -dynamic measures demonstrated a favorable bioavailability of the drug and effective metabolism of the substrates PPi and PLP [23]. In addition to biochemical testing, the early trials also recorded changes in disease manifestation, including survival, time on respiratory support and growth rate for severely affected pediatric patients, radiography for the assessment of bone mineralization and healing, a variety of functional tests for mobility, motor and muscle function, and patient reported outcomes (PROs) [23, 25, 27, 29]. These means remain important efficacy measures of asfotase alfa.

#### Pediatric

Two long-term studies, both initiated prior to approval of asfotase alfa, have recently reported important post-marketing evidence of the efficacy of asfotase alfa. Trial NCT01205152 comprised 10 children with perinatal or infantile HPP who were part of the initial phase 2 trial (NCT00744042) and followed them over 7 years [28]. The early improvements reported in 2012 [23] were sustained including improved skeletal mineralization, ongoing catchup growth in height and weight, respiratory function improvements that led to independence of respiratory support in all patients by four years of treatment, and notable ongoing improvements in developmental milestones including gross and fine motor function and cognition [28]. Data from the larger NCT01176266 trial in 69 children age 5 or younger reported that most (72%), but not all, infants/young children given asfotase alfa had early radiographic and clinical improvement that were sustained up to 6 years [27].

#### Adult

Data from a phase 2 trial (NCT01163149) provided initial evidence of sustained disease control in 19 (13 patients age 18 or older) pediatric-onset adolescents and adults treated with asfotase alfa [25]. Initiated in 2010 the study encompassed a primary treatment period of 6 months followed by an extension phase of 4.5 years. In contrast to the standard dose of 6 mg/kg/week used in almost all asfotase alfa studies, the first 6 month were dose at 2.1 or 3.5 mg/kg/week, followed by 6 months of 3.5 mg/kg/week and, at one year, start of the standard dose for the remaining 4 years of the study [25]. Pharmacodynamic data collected at 6 months and pooled from both initial dosings showed a statistically significant reduction in circulation PLP but not PPi in patients treated with asfotase alfa (n = 13) as compared to no treatment controls (n = 6) [25]. However, within subject data at study end revealed a significant reduction of both PLP and PPi compared to baseline [25]. Importantly, the reduced PPi level was within the lower normal range, thus limiting unwanted ectopic tissue mineralization. Despite the underdosing in the first year of treatment, functional improvements were observed. At 6 months, the 6-min walk test (6MWT) was significantly increased both in meters walked and percentage predicted for age; these improvements were sustained over the 5 year duration of the study [25]. Similar improvements have been previously reported in pediatric patients [24].

The subsequent phase 2 trial NCT02797821 further investigated asfotase alfa pharmacology in a cohort of 27 pediatric-onset adult patients. Pharmacodynamic data demonstrated that treatment with asfotase alfa normalized serum PPi, which is critical for improving bone mineralization, and also reduced the elevated level of PLP [31]. Importantly, the study confirmed the effectiveness of the standard dose of 6 mg/kg/week, which was developed mostly in children, for treatment of adults. Further, it confirmed maintenance of physiological PPi plasma levels in patients treated with asfotase alfa; notwithstanding, asymptomatic ectopic calcifications, in particular in the eye, have been repeatedly observed in HPP patients treated with asfotase alfa [24, 25, 31]. A twin report on pharmacokinetics revealed that the drug has a relatively long half-life of about 5 days, enabling less frequent dosing while maintaining stable therapeutic concentrations after about one months of treatment [30].

Three recent reports from the observational study NCT03418389 addressed bone mineralization and physical function. One report analyzed 21 adults with pediatric-onset HPP over 24 months [33]. Asfotase alfa treatment induced changes in bone turnover and mineral metabolism markers, suggesting that treatment-mediated mineralization may enable remodeling and bone turnover on previously unmineralized surfaces [33]. Another report recorded physical function in 22 patients over a period of 24 months [32]. Asfotase alfa treatment improved physical measures and reduced pain and fatigue [32]. A third report analyzed 14 pediatric-onset adult patients over 12 months and confirmed that asfotase alfa treatment enhances physical performance, as reflected in improved 6MWT and Timed Up and Go (TUG) scores, alongside better health-related QoL [34].

The therapeutic impact of asfotase alfa also includes substantial pain reduction and improvements in fatigue, key symptoms that significantly impair the daily lives of HPP patients. A recent real-world study collected PROs as part of a patient support program [40]. Data from 50 adults emphasized that over a period of 12 months patients experienced considerable decreases in pain levels and reported reduced fatigue, which improved their ability to perform daily activities [40]. Similar findings were recorded in a recent 3-year analysis of PRO data from 190 patients enrolled in the Global HPP Registry [12]. Patients reported lasting benefits in terms of reduced pain and disability, contributing to better QoL [12].

Data from PRO studies consistently underscore the improvements in QoL following asfotase alfa treatment. Findings from both the clinical study NCT03418389 and the patient support program reported improvements in physical and emotional well-being, reduced limitations in mobility, and enhanced participation in social and personal activities [34, 40]. Adult burden of illness data from the Global HPP Registry further highlighted the emotional and socioeconomic burden of HPP, emphasizing the importance of long-term management strategies like asfotase alfa therapy to alleviate these challenges [11].

Above evidence collectively highlight the significant therapeutic benefits of asfotase alfa in treating adults with pediatric-onset HPP, and span improvements in bone health, physical function, pain reduction, and overall QoL. Together, these studies validate asfotase alfa as an effective and safe therapy for managing pediatric-onset HPP in adults, offering substantial improvements in bone health, physical function, pain, and QoL. The sustained benefits of the therapy, alongside its favorable pharmacokinetics and safety profile, suggest that early and continuous treatment is crucial for mitigating the long-term complications of this rare disease.

#### Treatment Disruption and Lack of Efficacy

Enzyme replacement therapy for HPP is based on the continues supply of the missing TNSALP activity and, in theory, ceased supply results in reoccurrence of HPP symptoms. Recent data from study NCT01163149 proved this assumption. Six adult patients treated with asfotase alfa for 61–68 months but who then had an abrupt discontinuation of treatment for 15–48 months experienced the expected deterioration in clinical symptoms [26]. Upon resumption of asfotase alfa, they all showed clinical improvement underscoring the importance of treatment continuation [26]. However, not all retreated patients achieved improvements to treatment-naïve baseline levels, emphasizing the negative impact of treatment discontinuation on efficacy [26]. Lastly, it is important to keep in mind that not all asfotase alfa treated patients experience a meaningful and sustained treatment response. Perceived lack of efficacy is likely to contribute to treatment discontinuation in up to 10% of asfotase alfa-treated patients [41].

### Monitoring of Asfotase Alfa Therapy

#### Biochemical Testing

Changes in TNSALP substrates, such as PPi and PLP, are valid measures of the response to enzyme replacement and, as described above, have been utilized during the clinical development of asfotase alfa. Furthermore, urine PEA is an emerging clinical marker of HPP [42, 43]. However, in clinical practice determining these substrates often has limited value because of (a) lack of commercial PPi tests, (b) the potential inaccuracy of vitamin B6 (PLP) measures, for example due to continued metabolism in the test tube, and (c) the not fully developed clinical protocols for PEA assays. Therefore, physicians should refrain from titrating asfotase alfa dosing based on biochemical parameters. In addition, clinicians should keep in mind that accurate ALP testing depends on the selection of an appropriate reference range and that ALP testing on enzyme replacement therapy does not reflect endogenous enzyme activity. In fact, unexplained lowering of ALP levels while on enzyme replacement therapy may signal a compliance or an antibody issue and further evaluation may be warranted. Lastly and probably most important there is currently no data that demonstrates changes in biochemical parameters have any effect on outcomes.

#### Amended Guidelines

Comprehensive, evidence-based consensus guidelines for monitoring of asfotase alfa therapy were first published in 2017 [15]. Since then, clinical practice has adopted several important changes based on clinical observations. First, bone densitometry, specifically dual-energy X-ray absorptiometry (DXA), is not always a reliable marker of disease severity or bone health in individuals with HPP. In HPP, the quality of bone, particularly its mineralization, is often compromised despite the bone density readings potentially being normal or even elevated in some cases [44]. Data showed that both the quality of bone and its structural integrity, which contribute to fracture risk, are not adequately captured by DXA [45, 46]. Second, bone biopsies are not generally advised anymore because they may destabilize the already compromised HPP skeleton, thus increasing fracture risk [47]. Third, mobility in adult patients is now routinely measured using TUG and a Five Times Sit-to-Stand Test (5TSTS) in addition to the established 6MWT. Both TUG and 5TSTS are widely used in clinical assessments of balance, strength, and mobility in other musculoskeletal and metabolic conditions, including osteoporosis, and are recommended because the administrative burden for staff in the clinic setting is greatly reduced compared to the 6MWT. Lastly, PRO measures, such as PHQ9 or SF-23v2, have been recognized as important tools for clinical decision making [40]. Table 3 provides up-dated recommendations for monitoring asfotase alfa therapy in HPP patients.

**Table 3:** Up-dated Monitoring Guidelines for Patients With HPP Treated With Asfotase Alfa^*,^^§^

	Severe Perinatal/Infantile	Childhood	Adult
Assessment			
Craniosynostosis	Baseline, and every 3 months in first year, then every 6 months until age 3, or per neurosurgery Consult neurosurgery if suspected	Baseline and then annually Consult neurosurgery if suspected	n/a
Dental	Baseline, then once teeth erupt every 3–6 months as per dentist	Baseline, then every 6 months or as per dentist	Baseline, then every 6 months or as clinically indicated
Gastrointestinal Nutrition	Baseline, 3, 6 and 12 months and then annually as indicated High risk failure to thrive Frequent need for gastrostomy tube feeds	Baseline and then as clinically indicated	Baseline, then as clinically indicated
Gastrointestinal Nutrition	Baseline, 3, 6 and 12 months and then annually High risk failure to thrive Frequent need for gastrostomy tube feeds	Baseline and then as clinically indicated	Baseline, then as clinically indicated
Growth	Baseline length, weight, head circumference Evaluate every 3 months until age 4, and then every 6 months	Height and weight every 6 months	n/a
Hypersensitivity Reactions Injection Site Reactions Lipodystrophy	Each clinical assessment	Each clinical assessment	Each clinical assessment. Review of anaphylaxis risk by allergist
Lab Assessments			
- Baseline	CBC, CMP, PLP, Phos, 25-hydroxy vitamin D, PTH, urine Ca/Cr	CBC, CMP, PLP, Phos, 25-hydroxy vitamin D, PTH, urine Ca/Cr	CBC, CMP, PLP, Phos, 25-hydroxy vitamin D, PTH, urine Ca/Cr
- 1 months	CMP, Phos, 25-hydroxy vitamin D	CMP, Phos, 25-hydroxy vitamin D	CMP, Phos, 25-hydroxy vitamin D
- 3 months	CMP, Phos, 25-hydroxy vitamin D, urine Ca/Cr	CMP, Phos, 25-hydroxy vitamin D	CMP, Phos, 25-hydroxy vitamin D
- 6 months	CBC, CMP, Phos, 25-hydroxy vitamin D, urine Ca/Cr	CBC, CMP, Phos, 25-hydroxy vitamin D, urine Ca/Cr	CBC, CMP, Phos, 25-hydroxy vitamin D, urine Ca/Cr
- Annually	CBC, CMP, Phos, 25-hydroxy vitamin D, urine Ca/Cr	CBC, CMP, Phos, 25-hydroxy vitamin D, urine Ca/Cr	CBC, CMP, Phos, 25-hydroxy vitamin D, urine Ca/Cr
Mental Health	n/a	Screen each clinical visit Consider BASC-3, CSHQ, VABS-3, ADHD- RS	Baseline, 3, 6 and 12 months and then annually, Mental health specialist as needed Consider PHQ9
Motor Function Mobility Gait	PT/OT evaluation at baseline, 3, 6 and 12 months and then per therapist recommendation, or at least every 6 months until age 3. Then annually, or as indicated Consider BSID-III, PDMS-2, AIMS, GMFM	Baseline evaluation by OT/PT with 6MWT, TUG, 5TST If abnormal, thereafter at least every 6–12 months Manual motor/muscle testing as part of the physical exam and consider TUG, 5TST6MWT, TUG, 5TSTS (for ambulatory children ≥ 5 years) Consider BSID-III, PDMS-2, AIMS, GMFM	Baseline, 3, 6 and 12 months and then annually Manual motor/muscle testing as part of the physical exam Consider 6MWT, TUG, 5TSTS. As needed, physical therapy to aid test implementation Videotape gait and GAITRite at specialized centers
Nephrocalcinosis	Baseline, then every 3–6 months	Baseline, 6 months, and then annually	Baseline, then annually. Patients with hypercalcemia or hyperphosphatemia as needed
Pain	Baseline, then assess generally with each visit Consider NIPS	Baseline, then 6 months as indicated Consider Wong-Baker FACES Pain Rating Scale, 0–10 numeric pain rating scale, NIPS, CHAQ, PODCI	Baseline, 3,6 and 12 months and then annually Consider Wong-Baker FACES Pain Rating Scale, 0–10 numeric pain rating scale, BPI
QoL	Baseline, then assess every 6 months as indicated Consider PedsQL Infant Scales, EQ-5D-5L	Baseline, then every 6 months as indicated Consider PedsQL or Parent-Proxy Report, EQ-5D-5L	Baseline, 3, 6 and 12 months and then annually Mental health specialist as needed Consider EQ-5D-5L, SF-36(v2), HAQ, WPAI:SHP, PROMIS-29, RAPID3, TSQM
Radiography	Baseline bone survey Knees/wrist/chest 3, 6, and 12 months After 1 year, wrists annually and knees every 2 years, or as clinically indicated	Baseline knees, wrists, other sites as indicated If rickets, wrists and knees every 6 months until healed If no rickets, only as clinically indicated	Baseline and follow up as clinically indicated Screening for pseudofractures and insufficiency fractures as indicated
Respiratory	Critically important Baseline, then per pulmonologist Sleep study prior to hospital discharge Safety evaluation prior to airflight	Baseline evaluation and PFTs, then per pulmonologist ENT evaluation for OSA as indicated	n/a

^*^Devised from previously published guidelines [15]

^§^Based on resources available at a specialized HPP center. Care providers are encouraged to align these guidelines with their available resources and to seek outside expertise, for example physical therapy, or referral to specialized HPP centers as needed

*ADHD-RS* The ADHD Rating Scale-5, Home Version, *AIMS* Alberta Infant Motor Scale, *BASC-3* The Behavior Assessment System for Children, Third Edition, *BPI* Brief Pain Inventory, *BSID-III* Bayley Scales of Infant and Toddler Development, Third Edition, *ALP* alkaline phosphatase, *Ca/Cr* calcium/creatinine ratio, *CBC* complete blood count, *CHAQ* Childhood Health Assessment Questionnaire, *CMP* comprehensive metabolic panel, *CSHQ* The Children’s Sleep Habits Questionnaire, *EQ-5D-5L* EuroQol 5-Dimension 5-Level Health Questionnaire, *GMFM* Gross Motor Function Measure, *HAQ* The Health Assessment Questionnaire, *HPP* hypophosphatasia, *6MWT* 6-Minute Walk Test, *n/a* not applicable, *NIPS* Neonatal Infant Pain Scale, *QoL* quality-of-life, *OSA* Obstructive Sleep Apnea, *OT* occupational therapy, *PDMS-2* Peabody Developmental Motor Scales, Second Edition, *PedsQL* Pediatric Quality of Life Inventory, *PHQ* Patient Health Questionnaire-9, *Phos* inorganic phosphate, *PFT* pulmonary function test, *PLP* pyridoxal-5′-phosphate, *PODCI* Pediatric Outcomes Data Collection Instrument, *PODCI* Pediatric Outcomes Data Collection Instrument, *PPi* inorganic pyrophosphate, *PROMIS-29* Patient-Reported Outcomes Measurement Information System version 29, *PT* physical therapy, *PTH* parathyroid hormone, *RAPID3* Routine Assessment of Patient Index Data 3, *SF-36(v2)* Medical Outcomes Study Short Form-36 Health Survey (version 2), *TSQM* Treatment Satisfaction Questionnaire for Medication, *5TSTS* Five Times Sit to Stand Test, *TUG* Timed Up and Go Test, *VABS-3* The Vineland Adaptive Behavior Scales, Third Edition, *WPAI:SHP* Work Productivity and Activity Impairment Questionnaire

### Management of Long-term Treatment with Asfotase Alfa

#### Adverse Reactions

The prescribing information for asfotase alfa lists injection side reactions (ISRs) (63%), lipodystrophy (28%), ectopic calcification (14%), and hypersensitivity reactions (12%) as the most common side effects [22]. These incidences were based on approximately one hundred pediatric-onset HPP patients [22]. Recent data from the post-approval observational study NCT03418389 reported ISRs and lipodystrophy in 86% and 82% of 22 pediatric-onset adult patients, respectively [32]. A sub-group analysis of 14 patients from the same study found ISR rates of 79% and 93% after 3 and 12 months of asfotase alfa treatment, respectively, suggesting a temporal ISR increased [34]. Newer data from 216 HPP adults in the Global HPP Registry reported a rate of 12% ISR [12]. However, the stark difference between this rate and the ISR rates from clinical studies necessitate further validation of the real-world data.

Injection site reactions from asfotase alfa administration are generally transient and manifest as erythema, discoloration/hypopigmentation, pain/tenderness, pruritus/itching [22, 32]. Prevention of ISRs relies on rotation between 5 common injection sites [15, 22]. Management of patients prone to mild to moderate ISRs include administration of antihistamine plus acetaminophen or ibuprofen prior to asfotase alfa injection [15]. It is likely that there is an impact of ISRs on treatment adherence and that impact may increases with prolonged therapy, but data to support this notion is largely elusive.

#### Immunogenicity

Human anti-asfotase alfa antibodies were observed during early clinical development and the prescribing information for asfotase alfa states an anti-asfotase alfa antibody rate of 89%, with 57% of patients also tested positive for neutralizing antibodies (nAbs) [22, 23]. Recent studies NCT02797821 and NCT01176266 found anti-asfotase alfa antibody rates of 60% (n = 27) and 88% (n = 60), respectively [27, 31]. Despite the prevalence of anti-asfotase alfa antibodies, available data is limited. Study NCT01176266 provided the most detailed assessment. Consistent with previous observations, 67% of patients tested positive for nAbs over the course of a 6-year study [27]. No clear relationship was found between the presence of anti-asfotase alfa antibodies and adverse events nor were any adverse events suggestive of immune mediations [27]. It is well established, however, that nAbs can negate the clinical benefit of biologics, while non-neutralizing antibodies can also reduce efficacy, for example by affecting clearance, pharmacodynamics and pharmacokinetics [48]. Such adverse clinical effects are particularly relevant for long-term protein replacement therapy to treat monogenic disease [48]. Further investigation of the immune response elicited by asfotase alfa is needed and will aid mitigation strategies, such as dose escalation, immunomodulation, or asfotase alfa derivatives.

### Development of Efzimfotase Alfa

At the time of writing, physicians have gained about a decade of clinical experience with asfotase alfa. Clinical data demonstrates that most treated patients experience improved symptoms with manageable side effects. This raises the prospect of a lifelong asfotase alfa therapy and thus sustained control of HPP symptoms. However, asfotase alfa requires between 3 and 6 weekly injections which frequently induce ISRs. These characteristics challenge patient compliance not only in the pediatric population but also in patients requiring lifelong treatment. Therefore, asfotase alfa derivatives with optimized administration characteristics are under development. Proprietary modifications include TNSALP mutations for increased catalytic activity, alternate Fc regions, and variations in the bone targeting motif [49]. A lead compound, termed ALXN1850 or efzimfotase alfa, has entered development. The TNSALP domain of efzimfotase alfa lacks two N-linked glycosylation sites and harbors a single point mutation (E108M). In addition, it comprises the Fc part of human IgG2/4 instead of the Fc part of human IgG gamma-1, while maintaining the deca-aspartate bone targeting motif. In vitro, these modifications resulted in the expected increase in enzymatic activity [49]. The first clinical data from 15 adult patients treated with efzimfotase alfa became recently available [35].

Adults treated with efzimfotase alfa received either 15 mg (n = 5), 45 mg (n = 5) or 90 mg (n = 5) as a single intravenous injection followed by 3 weekly subcutaneous injections. Primary outcome measures were safety and tolerability, while secondary outcomes included pharmacokinetics and dynamics. Treatment-emergent adverse events occurred in 80% of patients and were related to treatment in 67% of patients [35]. Anti-drug antibodies were detected in 27% of patients [35]. Over the approximately 12 week course of the study, treatment-emergent adverse events were mainly related to ISRs and occurred in 40%, 40%, and 20% of patients dosed with 15, 45, and 90 mg efzimfotase alfa, respectively [35]. Although there currently is insufficient safety data for a robust side-by-side comparison between efzimfotase alfa and asfotase alfa, two previous studies in adults with pediatric-onset HPP have recorded treatment-emergent adverse events after 9 and 12 weeks of 6 mg/kg/week asfotase alfa [31, 34]. Both studies observed ISRs in 78% of patients [31, 34]. With respect to secondary outcome measures, the dose-adjusted and bodyweight-normalized plasma total exposure from efzimfotase alfa was approximately 21-fold higher after intravenous injection and 17-fold higher after subcutaneous administration when compared to asfotase alfa phase 1 trial data [35]. Together, these findings support the development of efzimfotase alfa as second-generation enzyme replacement therapy for HPP and have prompted initiation of phase 3 clinical testing in treatment-naïve pediatric patients (NCT06079359), in adolescent and adult patients who have not been previously treated with asfotase alfa (NCT06079281), and pediatric patients previously treated with asfotase alfa (NCT06079372).

### Alternatives to Enzyme Replacement Therapy

Discontinuation rates for asfotase alfa might be as high as 40% with most patients terminating treatment due to side effects or perceived lack of efficacy, and thus alternatives to enzyme replacement therapy are clinically relevant [41]. Because HPP is driven by a chronic reduction in TNSALP activity, drugs with an anabolic effect on TNSALP production have therapeutic potential, particularly in monoallelic patients with an intact copy of *ALPL*. The best characterized anabolic drug for bone is teriparatide, a recombinant protein containing the 34 N-terminal residues of human parathyroid hormone. Teriparatide can act as a potent stimulator of TNSALP production in osteoblasts and has been used for osteoporosis treatment since the early 2000s [50]. In 2007, it was first tested in an HPP adult [51]. The study found significant improvement in fracture healing and bone pain, suggesting a beneficial effect on bone remodeling [51]. These findings were corroborated in several case reports showing improved mineralization, fracture healing, long-term fracture reduction, and improvement in pain and QoL [52–55]. Together, these studies suggest that teriparatide offers benefits for adult HPP patients. However, efficacy appears variable [56], long-term effects on overall disease progression remain unclear [57], safety data are limited, and use in HPP remains off-label. The osteoblast inhibitor sclerostin has been investigated as an alternate anabolic target in HPP [58]. A total of 8 adult patients received 3 escalating doses of an anti-sclerostin antibody over the course of 5 weeks, followed by a 16 week observation period. Increased TNSALP was observed, but only during the dosing phase. Biochemical measures showed enhanced but transient bone formation [58]. The study, however, did not report on radiological findings, clinical manifestations or functional measures of HPP patients and thus from a clinical perspective remains preliminary. More recently an additional HPP case report suggested a positive effect of anti-sclerostin antibody treatment on insufficiency fractures [59]. In contrast to the other studies discussed in this article, both teriparatide and anti-sclerostin antibodies have only been investigated in case reports, case series, and a small clinical investigation. Clinical trials are still needed to establish definitive recommendations for HPP.

### Areas of Ambiguity in the Medical Treatment of HPP

Cases of patients presenting with HPP plus an additional suspected or confirmed metabolic bone disease are reported with increased frequency, highlighting the importance of a thorough evaluations to fully characterize the underlying mechanism of disease prior to treatment. For example, a recent study reported a family with a father and three children all with genetically confirmed HPP as well as osteogenesis imperfecta [60]. They presented with poor growth, multiple fractures, low bone mass but no rachitic disease. Based on bone biopsies which revealed severe osteomalacia, treatment was undertaken first with asfotase alfa followed by bisphosphonates, resulting in improved growth, increased bone mineral density and markedly reduced fracture incidence [60]. Less esoteric but more common is the general clinical conundrum presented among adults with HPP who are experiencing concurrent conditions resulting in osteoclast activation and pathologic bone resorption such as in oncology patients or post-menopausal deterioration of bone quality and bone density. Osteoclast inhibitors have been generally contraindicated in HPP due to concern for increasing the risk of atypical femur fractures in HPP. The approach to managing other skeletal disorders with co-existing HPP requires a careful individualized approach balancing the need for increased bone formation with decreasing bone resorption and longitudinal follow up to determine how the clinical course progresses.

## Summary and Outlook

Enzyme replacement therapy with asfotase alfa has revolutionized the treatment of HPP, bringing increased survival and improved functionality and QoL, although some minimally symptomatic patients may not require enzyme replacement. The last decade has seen several crucial developments in the medical treatment of HPP. First, long-term studies have demonstrated efficacy and safety of asfotase alfa up to 7 years. This is important because enzyme replacement is a chronic rather than curative treatment. Second, long-term treatment became a reality and prompted the development of a second-generation enzyme-replacement therapy for HPP. Third, large data sets on HPP became available. This now permits to more objectively quantify frequencies of HPP manifestations. Fourth, the value of real-world data and PROs became apparent. Long regarded as inferior to data collected in highly controlled clinical trials, PROs provide valuable insights in diseases such as HPP that are rare and considerably variable. Real-world PROs discovered, for instance, the prevalence of pain and chronic fatigue, in HPP adults. As underrecognized non-skeletal manifestations of HPP, such as pain or fatigue, become better understood, newer treatment modalities need to be developed to address these new phenotypes.

Key points for clinical practice are summarized in Box 3. Arguably a major challenge in clinical practice is the lack of evidence-based, consensus guideline for all steps of HPP care in both pediatric and adult patients. Current guidelines cover diagnosis and treatment monitoring, but not, for example, initiation of therapy or management of patients in which asfotase alfa is ineffective or contraindicated. Beyond clinical practice, guidelines recommending standardized tests for the clinical assessment of patients are needed as this will allow stratification of clinical observations. The authors urge for development of comprehensive guidelines for HPP care and clinical research.

All HPP patients merit timely diagnosis, treatment, and lifetime longitudinal follow-up for the assessment of disease progression. The expanding clinical experience with asfotase alfa begins to better define the treatment environment and long-term challenges. The authors remain concerned about timely access to appropriate care and therapy, partly because of the lack of awareness of HPP, the limited number of experienced specialists, the comparably high costs of asfotase alfa therapy, and especially the lack an approved enzyme replacement therapy for adult-onset patients in most countries. A broader approval of asfotase alfa for adult-onset HPP is supported by the documented efficacy in adults and the absence of unexpected safety signals in treated adults.

Despite its high therapeutic efficacy and good safety profile, it has become clear that asfotase alfa is not a one-fits-all therapeutic. For example, asfotase alfa is a cost-ineffective treatment for some HPP manifestations, such as isolated, modestly severe pain. Further, ISRs from asfotase alfa administration can limit or disrupt therapy. Moreover, although the underlying reason is not understood, asfotase alfa appears ineffective in a subset of patients. Therefore, cost-effective treatments with an alternate mechanism of action or route of administration have gained interest. Forward-looking preclinical studies have shown promise in using gene, cell-based, and mRNA therapies for HPP. For example, gene therapy, specifically with AAV vectors delivering the ALPL gene, improved survival and skeletal abnormalities in animal models [61], and cell-based therapy using engineered B cells has demonstrated sustained TNSALP production, improving in vitro bone mineralization [62]. Taken together, increasingly robust data suggests efficacy and safety of HPP enzyme replacement therapy independent of patient age and time of disease onset. Further improvements in the medical treatment of HPP can be anticipated.

### Box 3: Key Points for Physicians

1. Prevailing real-world symptoms of HPP include both skeletal and non-skeletal, manifestations across the lifespan. However, disease presentation is highly variable.
2. Education on HPP is important to recognize the disease early despite the often non-specific signs and symptoms.
3. Patients should be managed by an experienced HPP specialist.
4. Diagnostics of HPP should follow the established evidence-based consensus guidelines.
5. Enzyme replacement therapy, specifically asfotase alfa, can be a safe and highly effective treatment in patients of all ages and across symptoms.
6. Dosing of asfotase alfa should follow FDA recommendations.
7. No evidence-based consensus guidelines for initiation of asfotase alfa are available, but disease severity, genotype and manifestation provide guidance.
8. Disruption of asfotase alfa therapy in symptomatic patients should be avoided.
9. Monitoring of long-term therapy with asfotase alfa should follow established evidence-based consensus guidelines.
10. Intermittent PTH can be considered in adult patients when asfotase alfa is ineffective or contraindicated.

## Key References

- Whyte, M.P., et al., *Asfotase alfa for infants and young children with hypophosphatasia: 7 year outcomes of a single-arm, open-label, phase 2 extension trial.* Lancet Diabetes Endocrinol, 2019. **7**(2): p. 93–105.
  - This study showed the long-term efficacy and safety of asfotase alfa in HPP children.
- Pierpont, E.I., et al., *Impact of pediatric hypophosphatasia on behavioral health and quality of life.* Orphanet J Rare Dis, 2021. **16**(1): p. 80.
  - This study uncovered the behavioral health challenges in pediatric HPP.
- Rockman-Greenberg, C., et al., *Impact of discontinuing 5 years of enzyme replacement treatment in a cohort of 6 adults with hypophosphatasia: A case series.* Bone Rep, 2022. **17**: p. 101617.
  - This study showed the negative effect of disruption of asfotase alfa therapy.
- Dahir, K.M., et al., *Clinical profiles of treated and untreated adults with hypophosphatasia in the Global HPP Registry.* Orphanet J Rare Dis, 2022. **17**(1): p. 277.
  - Real-world data from the Global HPP Registry observed in HPP adults pronounced non-skeletal disease, including bone pain, muscle manifestations, dental problems and reduced QoL.
- Kishnani, P.S., et al., *Effectiveness of asfotase alfa for treatment of adults with hypophosphatasia: results from a global registry.* Orphanet J Rare Dis, 2024. **19**(1): p. 109.
  - This adult HPP real-world data set from the Global HPP Registry described lasting benefits from asfotase alfa in terms of reduced pain, disability, contributing to better QoL.
- Dahir, K.M., et al., *Safety, Pharmacokinetics, and Pharmacodynamics of Efzimfotase Alfa, a Second-generation Enzyme Replacement Therapy: Phase 1, Dose-escalation Study in Adults with Hypophosphatasia.* J Bone Miner Res, 2024.
  - This report described the first second-generation enzyme replacement therapy for HPP.

## Data Availability

No datasets were generated or analysed during the current study.

## Declarations

KD is a clinical trial investigator for Alexion, Regeneron, Ultragenyx, Kyowa Kirin, Sanofi. ND serves as a clinical trial investigator for Alexion and Ultragenyx, and as consultant and speaker board member for Alexion, Kyowa Kirin, and Biomarin.
